# Exploring the role of the Rab network in epithelial-to-mesenchymal transition

**DOI:** 10.1093/bioadv/vbae200

**Published:** 2024-12-14

**Authors:** Unmani Jaygude, Graham M Hughes, Jeremy C Simpson

**Affiliations:** School of Biology and Environmental Science, University College Dublin, Dublin, Ireland; Cell Screening Laboratory, School of Biology and Environmental Science, University College Dublin, Dublin, Ireland; School of Biology and Environmental Science, University College Dublin, Dublin, Ireland; School of Biology and Environmental Science, University College Dublin, Dublin, Ireland; Cell Screening Laboratory, School of Biology and Environmental Science, University College Dublin, Dublin, Ireland

## Abstract

**Motivation:**

Rab GTPases (Rabs) are crucial for membrane trafficking within mammalian cells, and their dysfunction is implicated in many diseases. This gene family plays a role in several crucial cellular processes. Network analyses can uncover the complete repertoire of interaction patterns across the Rab network, informing disease research, opening new opportunities for therapeutic interventions.

**Results:**

We examined Rabs and their interactors in the context of epithelial-to-mesenchymal transition (EMT), an indicator of cancer metastasizing to distant organs. A Rab network was first established from analysis of literature and was gradually expanded. Our Python module, *resnet*, assessed its network resilience and selected an optimally sized, resilient Rab network for further analyses. Pathway enrichment confirmed its role in EMT. We then identified 73 candidate genes showing a strong up-/down-regulation, across 10 cancer types, in patients with metastasized tumours compared to only primary-site tumours. We suggest that their encoded proteins might play a critical role in EMT, and further *in vitro* studies are needed to confirm their role as predictive markers of cancer metastasis. The use of *resnet* within the systematic analysis approach described here can be easily applied to assess other gene families and their role in biological events of interest.

**Availability and implementation:**

Source code for *resnet* is freely available at https://github.com/Unmani199/resnet

## 1 Introduction

Rab GTPases are small GTP-binding proteins that regulate the transport of proteins, lipids, and various macromolecules between different compartments within the cell. They are activated and deactivated by GEFs (Guanine nucleotide exchange factors) and GAPs (GTPase-activating proteins), respectively, to alternate between a GTP-bound active and a GDP-bound inactive state. When active, they bind to specific subcellular membranes and recruit effector proteins, which in turn play various roles in the transport of cargo molecules. With over 60 members identified in humans, Rabs, along with these accessory proteins, perform a critical role in intracellular membrane trafficking (Homma *et al.* 2021). Furthermore, mutations in several Rab GTPases have been implicated in a range of human diseases including a number of cancers (Jin *et al.* 2021). The interaction network of Rab GTPases, along with their GEFs, GAPs, and effectors, has been previously termed the ‘*Rabome’* (Gurkan *et al.* 2005, Pinar and Peñalva 2021). As further interactors are identified, this revised *Rabome* will inevitably grow in size. We therefore adapt this term within our study, allowing expansion of the *Rabome* to include a wider set of interacting proteins, providing a systems level understanding of how it operates and contributes to disease progression.

In addition to their master role in intracellular membrane transport, some Rab proteins (RAB5, RAB6, RAB7, and RAB21) function in essential processes related to cellular motility such as cell-cell adhesion, migration, and regulating the cytoskeleton during cell migration (Tang and Ng 2009, Mendoza *et al.* 2014, Margiotta *et al.* 2017, Vestre *et al.* 2019). Their role in governing cellular motility makes the interaction network of Rabs, or *Rabome*, an attractive network for studying epithelial-to-mesenchymal transition (EMT) in cancer cells. During EMT, epithelial cancer cells change to mesenchymal type, suppressing their epithelial features and gaining mobility. EMT marks the beginning of metastasis by allowing the primary tumour to break off and migrate to distant organs through blood and/or lymphatic system (Yao *et al.* 2011). This cellular reprogramming also induces stemness in cancer cells, in addition to the migratory phenotype (Kong *et al.* 2010, Caja *et al.* 2011, Rhim *et al.* 2012). Stemness characteristics, such as self-renewal and proliferative potential, sustain the cancer progression resulting in cancer relapse and chemoresistance (Phi *et al.* 2018). Although there are some links between Rab GTPases and the stemness status of cancer cells (Brunel *et al.* 2021), RAB35, RAB21 and RAB5A proteins have been shown to promote cell migration by altering the recycling of β1 integrins leading to cancer metastasis (Pellinen *et al.* 2006, Argenzio *et al.* 2014). As recurring tumours remain a major concern, despite combinatorial cancer treatments (Li *et al.* 2015, Lim *et al.* 2022), improving the diagnosis of metastasis is a key step in treatment. Patients may get a recurring tumour from the traces of mesenchymal cancer cells in the lymphatic system settling to distant organs even after successful surgery removal of a tumour. Hence, it is important that patient screening and monitoring methods include testing for biomarkers of EMT.

Here, we employ an *in silico* approach to explore the interaction network of Rab GTPases to identify such predictive markers of EMT linked to the following cancer types—urinary bladder, breast, cervix, oesophagus, kidney, liver, lung, prostate, stomach, and thyroid. We demonstrate the utility of protein–protein interaction networks (PPINs) in our study and have developed *resnet*, a Python-based module, to assess the resilience of the expanded Rab PPINs. Using *resnet*, we select an optimally sized and resilient Rab interactome for further analyses using the MaxLink approach (Ostlund *et al.* 2010). Finally, amongst the 73 candidates identified as hits, we suggest RAB3B, RAB6B, and RAB40A proteins may have a crucial role in cancer metastasis. Examining Rabs within a network-based framework (the Rab interactome) demonstrates the utility of our holistic approach in providing both a comprehensive view of target protein families and in understanding complex mechanisms such as EMT. Our network-based approach provides a method for combining PPIN-based data with mRNA expression data to improve metastatic diagnoses.

## 2 Methods

### 2.1 Construction of basic Rab PPIN

A PPIN of Rab GTPases was formed from the interactions compiled and reviewed by Homma *et al.* (2021). This PPIN consisted of a total of 421 proteins (nodes), which were made up of 59 Rabs and their 362 interacting nodes. Across this network, a total of 734 interactions (edges) were identified. The base network was visualized in Cytoscape v3.9.1 (Shannon *et al.* 2003). An additional 1104 edges amongst the non-Rab proteins that were not listed in the review Homma *et al.* (2021), were added from STRING database v12.0 (Szklarczyk *et al.* 2023) using StringApp (Doncheva *et al.* 2019). This 421-node network acted as the ‘base’ network and was further expanded serially.

### 2.2 Selecting the optimal *rabome*

The base network was repetitively expanded 23 times by adding nodes with a high confidence score (>0.7) from all STRING channels. The confidence score denotes the probability that the interaction exists between two nodes (Szklarczyk *et al.* 2023). The base network and all the serially expanded 23 PPINs were treated as undirected and binary networks. The general topological properties of all Rab PPINs were determined by the Cytoscape plugin Analyze Network (Assenov *et al.* 2008).

Determining network resilience is useful in optimizing network-like systems for efficiency and robustness in various fields (Dunn & Wilkinson 2016, Herrera *et al.* 2016, Drakopoulos & Mylonas 2020). It is a measure of robustness of systems to random breakdowns or in this case, functional failure of proteins in the network resulting in pathogenesis or even cell death. The modified Shannon diversity index introduced by Zitnik *et al.* (2019) was used to measure the resilience of all PPINs by inducing random node failure. The base network was expanded and was assessed for resilience with the purpose of selecting the most optimal Rab PPIN or the ‘optimal *Rabome’* for further analyses.

The network resilience of the base network and 23 Rab PPINs of increasing sizes was measured. The expansion of the base network occurred alongside measuring the resilience of the resulting PPINs. The base network was expanded until the resilience stopped increasing and was stabilized over time. Initially, the base network was expanded by only 50–100 nodes, which showed increasing resilience until a certain network size. After this, larger increments (∼500 nodes) were done until the increase in resilience had minimized and plateaued. For this purpose of assessing resilience, we developed a Python module *resnet* (https://github.com/Unmani199/resnet). Resilience was calculated using the modified Shannon diversity index (H_msh_) that accounts for the rising Entropy (Eq. 2) in the system upon deletion of random nodes at an increasing failure rate, f.

(1) $$\mathrm{Failure}\;\mathrm{rate}\;\left(f\right)=\frac{\mathrm{Nodes}\;\mathrm{removed}}{\mathrm{Total}\;\mathrm{nodes}}\;$$

 

(2) $$\text{Entropy}\;(H_{\text{msh}})=-\Sigma_{i=0}^{k}\;\frac{Ci}{N}\;\mathrm{Log}\frac{Ci}{N}$$

 

(3) $$\text{Resilience}=1-H_{\text{msh}}$$

*N* is the network size, *f* is the failure rate (Eq. 1), or the fraction of nodes removed and *k*, *C_i_* are the number and size of the components formed for each failure rate, respectively. *f* ϵ [0, 1] denotes the decay of the fully intact Rab network starting from *f *=* *0 to a completely defragmented network (*f *=* *1). The chaos or entropy (H) in the system also progresses from 0 to 1, denoting complete defragmentation of the network when the number of all components (Σ *C_i_*) equals the number of total nodes (N). To obtain a steady value of H for each failure rate, 500 iterations of random removal of nodes were performed. All entropy values (H) were plotted for each *f*, where the H_msh_ amounted to a cumulative rise in entropy or the area under the curve. By contrast, the resilience (Eq. 3) was measured from the area above the curve as (1 - H_msh_). The value of resilience ranges from 0 to 1, with 0 denoting an intact network and 1 being a completely fragmented network. The resilience values for all 24 Rab PPINs were calculated and normalized in the range of [0,1] (Supplementary File F1 Section S1). The smallest stable network from the resilience curve was used for further analyses to avoid the complexity potentially introduced by larger networks.

Measuring resilience for larger networks with *resnet* became time-consuming as the number of failure rates increased with network size. Hence, we divided the range of *f* ϵ [0, 1] into 100 and 200 bins, to successfully improve the computing time and efficiency of *resnet* (see Supplementary File F1, Section S3 for details on *resnet* testing). Based on the resilience values obtained by this method, the optimal Rab PPIN was selected, which we refer to as the optimal *Rabome*.

### 2.3 Topological analysis and pathway enrichment of the optimal *rabome*

The optimal *Rabome* found from the resilience testing was analysed for pathway enrichment, with FLAME (Karatzas *et al.* 2023) v2.0 (web application). Four pipelines, namely aGOtool, g: Profiler, WebGestalt, and Enrichr, were used in combination to identify enriched pathways from the KEGG (Kanehisha and Goto 2000), WikiPathways (Slenter *et al.* 2018), Reactome (Fabregat *et al.* 2018), and PANTHER (Mi *et al.* 2021) databases. The enriched pathways were tested for significance based on a combined P-value from the integrated use of the four pipelines. The significantly enriched pathways were examined further for involvement in EMT. Input parameters for FLAME are available in Supplementary File F1 Section S4. Topological properties of all 24 networks were obtained using the Cytoscape plugin ‘Analyze Network’ and were min-max normalized (Mazziotta and Pareto 2022).

### 2.4 Identifying proteins contributing to EMT

#### 2.4. 1 MaxLink approach

As a first step in the MaxLink approach (Ostlund *et al.* 2010), the optimal *Rabome* was assessed for proteins with established links to EMT. Such proteins were categorized as EMT-proteins due to their contribution to EMT, as found from dbEMT v2.0 (Zhao *et al.* 2019), a manually curated database of experimentally verified information. In the next step of MaxLink analysis, the immediate interacting nodes of the EMT-proteins were labelled as candidates and were put through two further filters: annotation and connectivity. Under the annotation filter, the Disease Ontology (DO) IDs for each candidate were obtained from OBO foundry (Jackson *et al.* 2021). Candidates were removed if the DO Identifiers (DOIDs) associated with them contained cancer-related terms (e.g. ‘glioma’, ‘cancer’, ‘tumor’, ‘cancer_DO_slim’). After the annotation filter, the remaining candidates were subsequently filtered based on their connectivity or degree. Candidates with a higher connectivity to EMT-proteins and an overall high degree were eliminated. We plotted the connectivity of candidates to EMT-proteins against their overall degree in the Rab network with a Loess fit (Supplementary File F1 Section S5). The candidates within the 95% CI of this fit were then eliminated.

#### 2.4. 2 Importing cancer expression data

After the annotation and connectivity filters, we analysed the mRNA expression of the finalized candidates from MaxLink analysis across 10 cancer types (with TCGA study abbreviations) - urinary bladder (BLCA), breast (BRCA), cervix (CESC), oesophagus (ESCA), kidney (KICH, KIRC), liver (LIHC), lung (LUAD), prostate (PRAD), stomach (STAD), and thyroid (THCA). The mRNA expression data for cancer patients across 10 cancers were obtained from TCGA datasets (https://www.cancer.gov/tcga). The ‘normal’ or non-tumorigenic expression was obtained from the GTEx dataset (Ardlie *et al.* 2015) for 10 tissues corresponding to their cancer counterparts. Wang *et al.* (2018) unified the GTEx and TCGA datasets by normalizing and batch-correcting the raw FPKM values from both studies. These normalized values for the aforementioned cancer types were used in our studies. Additional ‘normal’ tissue expression data from the TCGA dataset were used. Further details on the dataset sizes can be found in Supplementary File F1 Section S6 and the TCGA IDs for primary-site and metastatic patient samples in Folder F5.

#### 2.4. 3 Classifying patient sample data

Patient samples were classified as ‘primary stage’ and ‘metastasized stage’ based on the TNM cancer staging system (Rosen and Sapra 2023). In this system, primary stage (N0, M0, any T) indicates the presence of tumour only at the primary site and not in nearby lymph nodes and distant organs; late stage (N1/2/3 and any M) indicates the definitive spread of tumour in the nearby lymph nodes and/or to the distant organs to varying extents depending on M. Patient samples with missing data within any part of the TNM code (TX, NX, MX) were not included. All cancer types were thus classified into two stages, primary and metastasized.

#### 2.4. 4 Analysis of differential expression

For each gene, whether an EMT gene or a candidate, a mean mRNA expression in FPKM (Fragments Per Kilobase per Million mapped fragments), was calculated for each cancer type within 3 cohorts as follows: normal tissues (FPKM_Norm_), primary-site tumour tissues (FPKM_Prim_) and metastatic tumour tissues (FPKM_Met_). The change in expression of each gene was measured using Fold Change (FC) for two transitions—normal tissue-to-primary site tumour and primary site-to-metastatic tumour. The FC for each gene is the ratio of its changed mean expression during the transition (e.g. mean FPKM_Met_/mean FPKM_Prim_). A Kruskal–Wallis (KW) test was done to assess the significance of each FC for both transitions. Lastly, candidates with high significance and medium significance were identified as hits and examined further.

## 3 Results

### 3.1 Expansion of the base Rab network

**Figure 1.**
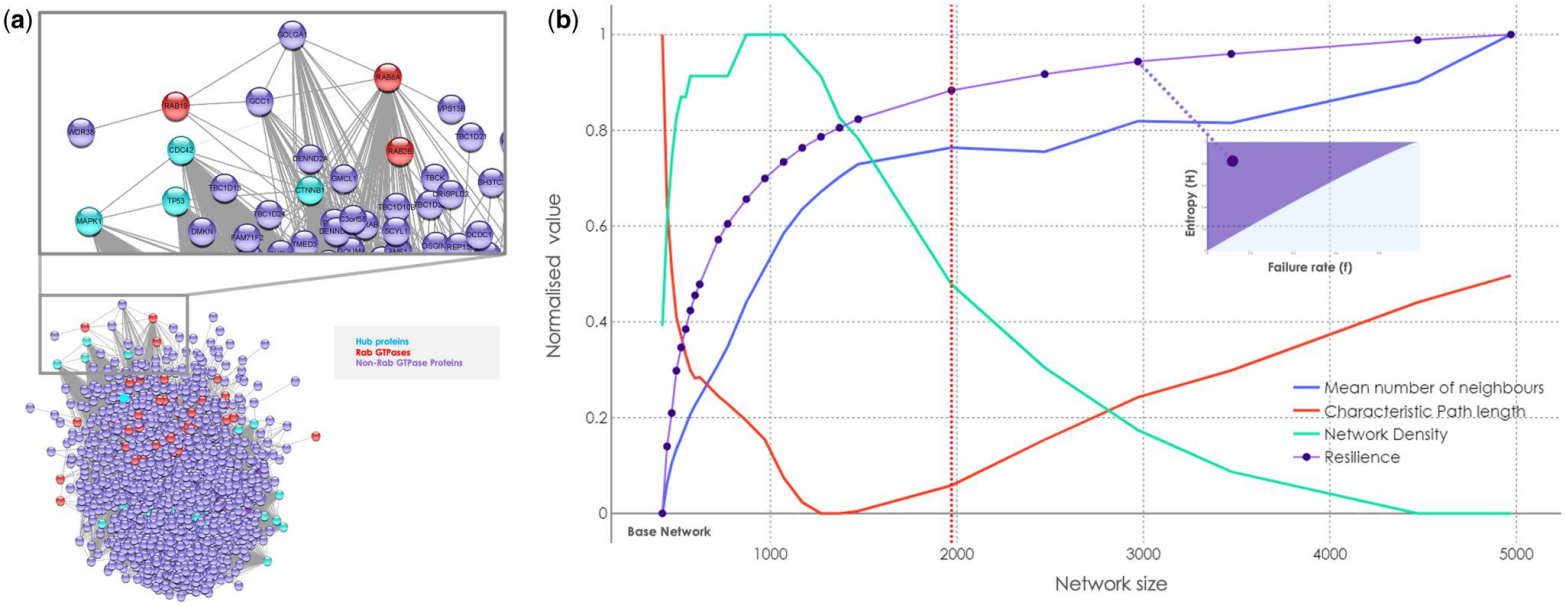
(**a**) The optimal Rab network visualised in Cytoscape. The nodes are colour-coded for hubs (cyan), Rab GTPases (red) and all other proteins (purple). See all the nodes and edges in Supplementary File F6. (**b**) The changing topological properties of Rab interactomes during the gradual expansion of the base network from 421 nodes to 4971 nodes. The inset shows the source of resilience values, i.e. the total area above the curve of the resilience plot generated by *resnet*. The red vertical line represents the ‘optimal’ network size of the optimal *Rabome* used for further analyses.

After the expansion of the base network established from a literature review, all PPINs were visualized in *Cytoscape* and their topological properties were determined (Fig. 1a). The general topology of the optimal *Rabome* altered when the base network was serially expanded from 421 nodes to 4971 nodes (Fig. 1b) (the topological properties for all Rab PPINs are provided in Supplementary File F1 Section S1). The average number of neighbours, clustering coefficient, and resilience rose with the network size, whereas the network density peaked at size = 1071 and had a sharp decline after. Characteristic path length (CPL), on the other hand, was the least at this network size and gradually increased during further expansion. CPL is the average number of nodes needed to be traversed to go from any one node to the rest of the nodes in the network. Hence, it is analogous to the shortest path length between two nodes. For example, in Fig. 1a, GOLGA1 and GCC1 are the two effector proteins (blue edges) or interacting nodes (dark grey edge) of RAB19 and RAB6A (Sinka *et al.* 2008). Due to no direct interaction between the two Rabs, their shortest path length is 1. Hence, a decreasing CPL indicates smaller shortest path lengths and growing compactness of the optimal *Rabome.* The apparent inverse relationship of CPL with network density may be due to denser networks generally having shorter paths due to most nodes being more interconnected allowing faster intra-node communication.

### 3.2 Selecting the optimal *rabome*

The resilience values for each network were obtained from the plots generated by *resnet*. Each of these plots (Fig. 1b inset) represents the rising entropy (H) in the system because of increased node removal or the failure rate, *f*. Starting from an intact network (H = 0, resilience = 1) until the complete defragmentation of the network (H = 1, resilience = 0), the area under the curve accounts for the total rise in entropy of the system. By the complementary relationship of entropy with robustness, the area above the curve denotes the resilience of the PPIN. The normalized resilience values appeared to stabilize for larger networks (>1500 nodes) in a steady state within the range of 0.8–1 (Fig. 1b). In other words, the network resilience did not increase to any great extent for networks larger than 1500 during the expansion. This can be confirmed by the minimal change (< 7%) in the normalized resilience for the networks in the steady zone (Supplementary File F1 Table 2). Hence, we termed the Rab PPIN of size 1971 ‘optimal’ since it was the smallest network within the steady zone and more suited for further analyses owing to better computational efficiency in comparison to the larger networks. Moreover, the CPL and network density converge not too far from this network. Hence, this is a less dense network with a reasonable characteristic path length making it a more suitable Rab PPIN to explore pathways.

### 3.3 Degree distribution and hubs of the optimal *rabome*

**Figure 2.**
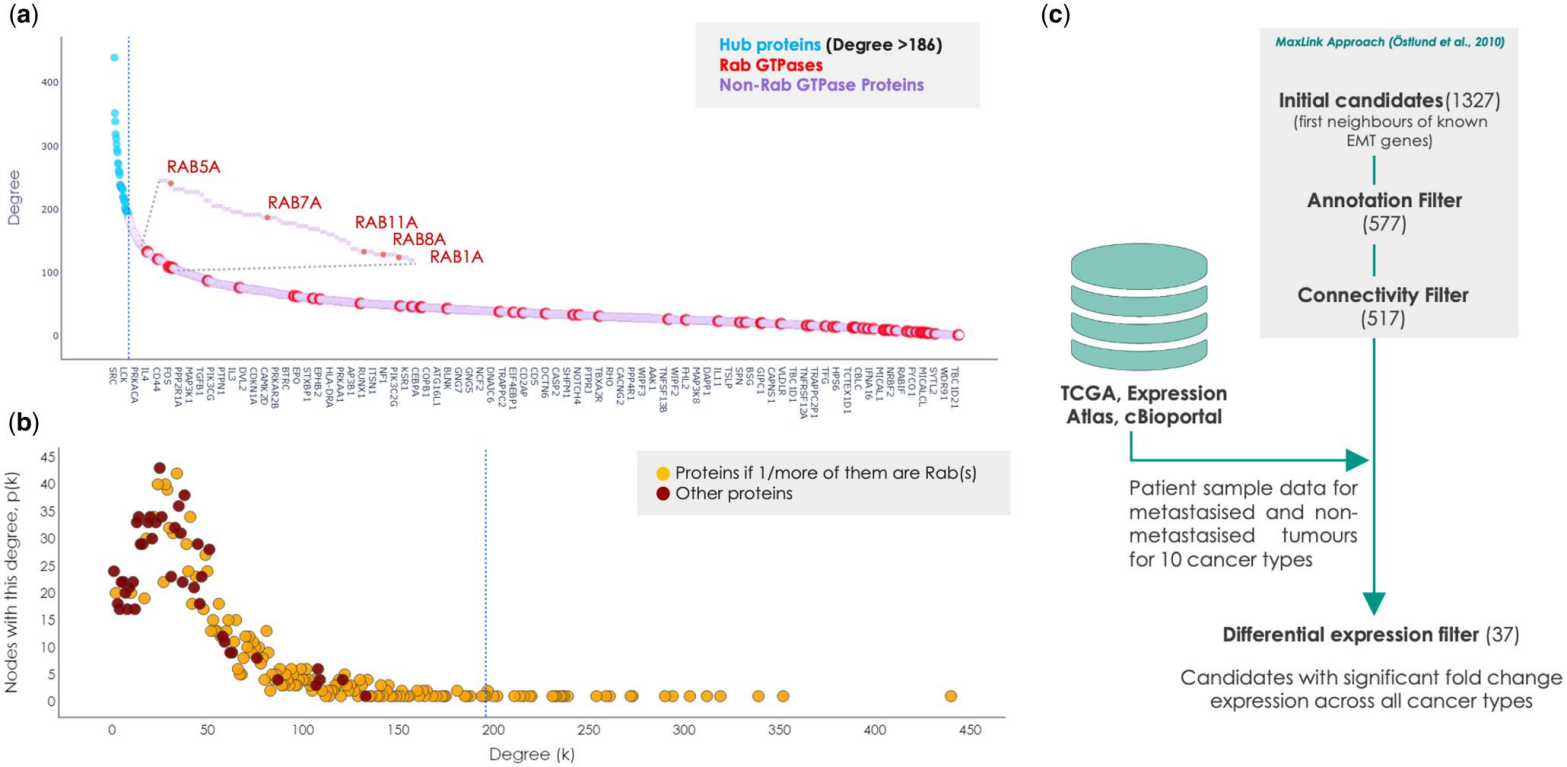
(**a**) The degree plot of the optimal Rabome with the elbow of the curve highlighting the hubs. Colour-coded for hubs (blue), Rab GTPases (red) and all other proteins (lilac). (**b**) The degree distribution of the optimal Rabome. Hubs identified by the elbow threshold in (**a**) are separated by the dotted blue vertical line on the X-axis at 188 nodes, i.e. k = 188. The Y-axis denotes the count of nodes with a degree k and is colour-coded maroon if one or more of the nodes are Rab(s). (**c**) Steps in MaxLink analysis used to identify EMT genes, their immediate neighbours (candidates), and consequent filters to identify candidates instigating the EMT.

From the optimal Rab network, we identified 33 hubs based on the elbow of the degree plot (Fig. 2a) using the kneedle algorithm (Satopaa *et al.* 2011). The top 10 proteins of the 33 hubs (with number of connections) were SRC (440), EGFR (352), AKT1 (339), HRAS (319), PIK3R1 (312), PIK3CA (303), GRB2 (294), MAPK3 (290), CTNNB1 (273), MAPK1 (272). In the base network, all the hubs consisted of Rabs since the network was established with a focus on Rab GTPases. However, with the network expansion, the overall number of connections of Rab GTPases dropped (Fig. 2a). In the optimal *Rabome*, Rab GTPases appear evenly spread over the degree plot, exhibiting a wide range of connectivity. The 5 topmost connected Rab GTPases were RAB5A (133), RAB7A (121), RAB11A (109), RAB8A (108) and RAB1A (107). All hubs with their degrees are detailed in Supplementary File F1 Section S2.

The fat-tailed degree distribution of the optimal *Rabome* resembled a weak scale-free structure (Fig. 2b). Scale-free networks follow the power law and tend to have many nodes with less connectivity while a few hubs monopolize the connections. The base network and all the expanded Rab networks weakly followed the power law p(k) = k ^-γ^, where k = degree, p(k) = number of nodes with the degree k, and the exponent γ ϵ (2, 3) for highly scale-free networks. For all the Rab networks γ lay between 1.2 and 1.3, which is consistent with the fact that strongly scale-free networks are rare across biological, digital and transportation systems (Broido and Clauset 2019). Depending on the extent of its scale-free nature, a network can be vulnerable to the intentional failure of hubs but quite robust to random failures (Goh *et al.* 2002, de Sol *et al.* 2006). Therefore, weak or not, the presence of a scale-free structure in Rab networks might explain the resilience shown by the larger Rab networks (Fig. 1b).

### 3.4 Enrichment analysis

We found 504 pathways significantly enriched in the optimal *Rabome* from the KEGG (Kanehisa and Goto 2000), WikiPathways (Slenter *et al.* 2018) and Reactome (Fabregat *et al.* 2018) databases. The enriched pathways were identified with the combinatorial use of the four distinct pipelines implemented within FLAME v2.0. Pathways were considered significantly enriched if, regardless of their source database, they had a high significance by all four pipelines with a combined P-value < 0.001. All enriched pathways are listed in Supplementary File F2. Eight pathways enriched in the optimal *Rabome* have been previously linked to the EMT such as NOTCH (Deshmukh *et al.* 2021), NOTCH3 (Ohashi *et al.* 2011, Liu *et al.* 2014), TGFβ (Ozdamar *et al.* 2005, Hao *et al.* 2019), Wnt, Hedgehog (Taipale and Beachy 2001), EGF/EGFR (Misra *et al.* 2012), FGFR (Ruan *et al.* 2023), and PI3K/AKT (Maharati and Moghbeli 2023) (Supplementary File F1 Section S4). No Rabs were found within these eight pathways except for RAB6A in NOTCH signalling and RAB23 in Hedgehog signalling.

### 3.5 MaxLink approach for identifying EMT-related hits

After confirming the involvement of the optimal *Rabome* in phenotype(s) associated with EMT, we followed the MaxLink approach, introduced by Ostlund *et al.* (2010), to identify novel genes potentially contributing to a phenotype from a network of genes already known to instigate that phenotype. Here, we adapt and modify the MaxLink analysis to identify genes with novel associations to EMT, based on their encoded proteins from the optimal *Rabome*. Using this approach, we identified proteins in the optimal *Rabome* previously known to instigate EMT and labelled their immediate neighbours ‘candidates’. These candidates are likely to be in the up/downstream of the EMT pathways, being the immediate neighbours of ‘EMT genes’. However, to identify any novel contribution of the candidates towards EMT, we eliminated those with previously known links to our target phenotype, EMT, e.g., candidates associated with cancer incidence. The candidates obtained downstream of MaxLink filters were then reviewed for their novel role in EMT.

#### 3.5. 1 MaxLink analysis

As a first step within MaxLink approach (Fig. 2c), we termed 346 genes as EMT genes due to their experimentally verified links to EMT as found in dbEMT v2.0 (Zhao *et al.* 2019). Their immediate interacting nodes were termed as candidates and put through 2 sequential filters. During the annotation filter, the candidates associated with cancer-related Disease Ontology terms were filtered out. 577 candidates after the annotation filter were subjected to the connectivity filter (see methods, Supplementary File F1 Section S5). We did this to avoid any pseudo correlation of such candidates to EMT. That is, a candidate could have many neighbouring EMT genes simply due to its higher overall connectivity within the optimal *Rabome*. For the remaining 517 candidates, we studied their mRNA expression in normal tissues and tumour samples across 10 cancer types.

#### 3.5. 2 Analysis of differential expression of candidates and EMT genes

**Figure 3.**
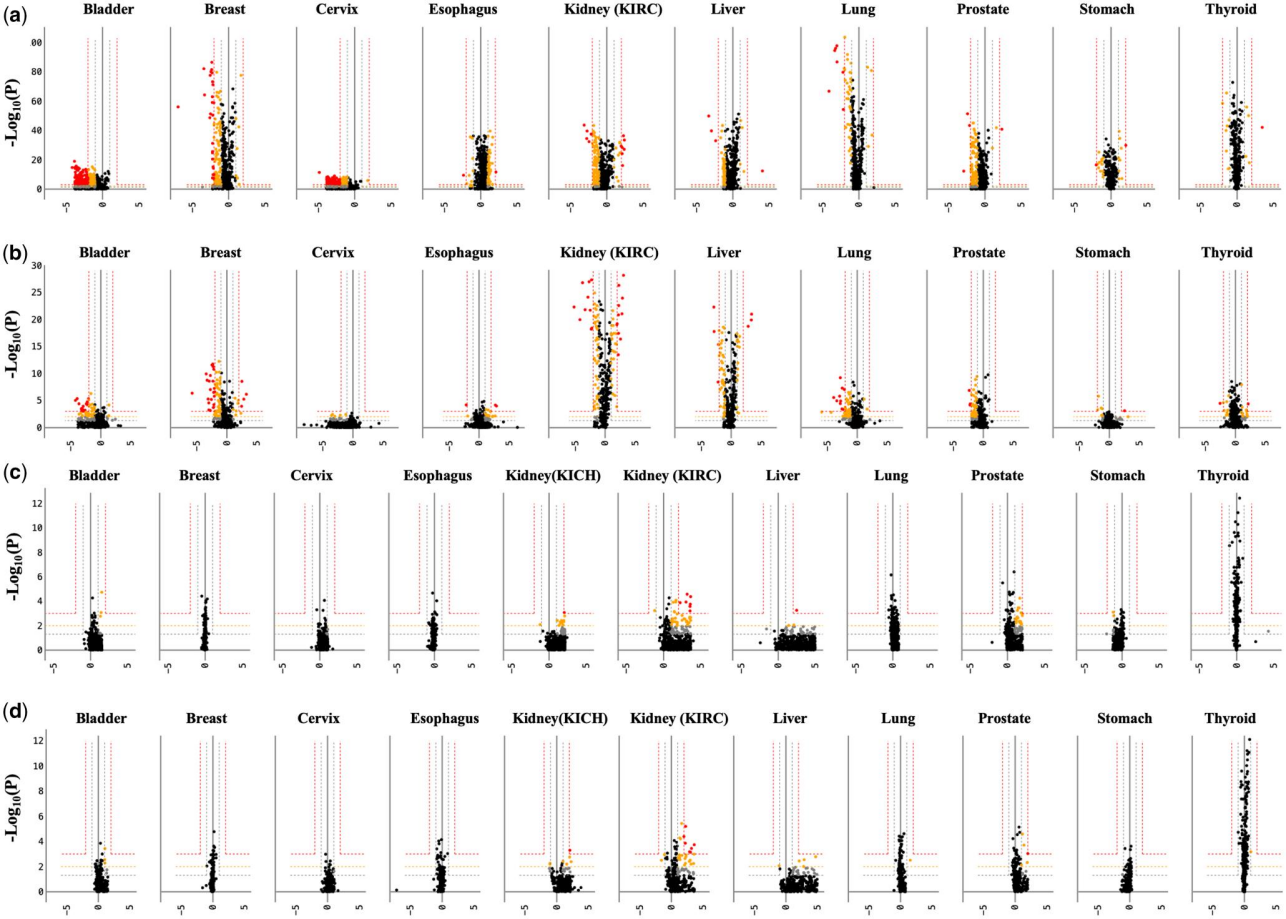
Log2FC in the expression (FPKM) of genes across 10 cancer types during two transitions—normal tissues to primary-site tumour (**a** Candidates, **b** EMT genes) and primary-site to metastatic tumour (**c** Candidates, **d** EMT genes). The high significance outer line (red) indicates genes with |log_2_FC|>2 and *P *< .001, followed by the medium significance line in orange (1<|log_2_FC| <2 and 0.001<*P *< .01), and the low significance innermost line in grey (1<|log_2_FC| <2 and 0.01<*P *< .05. Non-significant genes are in black (*P* > .05).

We assessed the change in expression levels of candidates as well as EMT genes for two transitions—normal tissue to primary-stage cancer and primary to metastatic-stage cancer. A notable difference in the differential expression of candidates as well as EMT genes during both transitions was seen. In particular, we observed a stronger signal in the normal-to-primary-stage transition than in the primary-to-metastatic stage transition (Fig. 3a–d). The number of statistically significant genes, both EMT genes and candidates, was higher during this transition, demonstrating a greater level of up-/down-regulation during cancer incidence across all 10 types.

We tested the significance of this differential expression using the KW test, considering the inconsistent cohort size of normal, primary-site and metastatic samples for some cancers (Supplementary File F1 Section S6). No minimum threshold was applied to the size for all three cohorts for two reasons. First, although the occurrence of some cancer types, such as KICH (kidney), was low, their inclusion was deemed essential for a well-rounded study. Secondly, the KW assessments were independent of cancer type and tested the significance of differential expression, accommodating for the smaller cohorts. We stratified the levels of significance within genes based on their Fold Change (FC) and KW significance (P-value) as follows—high (|log_2_FC| >2 and *P *< 0.001), medium (1<|log_2_FC| <2 and 0.001<*P *< 0.01), and low significance (1<|log_2_FC| <2 and 0.01<*P *< 0.05). Genes with *P *> 0.05 were considered non-significant. Expression data for ‘normal’ tissue samples was obtained from the GTEx and TCGA datasets, normalized and batch-corrected by Wang *et al.* (2018). We reanalysed the normal to primary-site transition using the GTEx and TCGA datasets individually in addition to their combined use presented so far. We did not observe a major difference (<10%) in the Log2FC of genes, upon the individual use of GTEx and TCGA-Normal samples (Supplementary File F3) rather than combined. Due to a low number of normal tissue samples, KICH (kidney) was not assessed for normal to primary transition (Fig. 3a–d). Being a rare type of renal cancer compared to the more commonly occurring KIRC, we only assessed KIRC for normal to primary transition.

Finally, we identified the candidates with a significant up-/down-regulation during the progression of metastasis, i.e. the transition from primary-stage to metastatic-stage cancer. In order to do this, those with high and medium significance were selected and labelled as hits (Table 1). Of the 73 hits identified, nine had high significance and 64 of them appeared with a medium significance in at least one cancer type (Supplementary File F4). Over half of the total hits showed a differential expression in two types of kidney-related cancers—kidney chromophobe (KICH) and kidney renal clear cell carcinoma (KIRC). The following gene hits appeared with high significance—*SORBS1, SIKE1, RAB3B, NCF1, LRP8, IL27, FGF5, EBI3, BAIAP2L1*. Six gene hits appeared in kidney as well as prostate cancers (Table 1)—*IL27*, *ZWINT*, *LY96*, *LAT*, *IGLL5*, and *AKAP5.* The final hits, namely the 73 genes from the optimal *Rabome*, can be further explored for patterns in their mRNA deregulation and the possible malfunctioning of their encoded proteins, leading to cancer metastasis.

**Table 1.** Hits and the metastatic cancer(s) they demonstrated altered gene expression in.

Cancer Type	Number of hits	Hits
Kidney (KIRC)	39	**RAB3B**, **NCF1**, **LRP8**, **IL27**, **FGF5**, **EBI3**, **BAIAP2L1**, RAB6B, ZWINT, WAS, UBE2D1, SLC12A5, SH2B2, SEC24D, RIPK2, RILPL1, RHOQ, RFFL, PSENEN, MYRIP, MAP1LC3C, LY96, LILRB2, LBP, LAT, LAMTOR2, IL37, IGLL5, GNG4, GNA15, FCGR1A, CSF2RB, COPG2, CD72, CARD9, AKAP5, AGTRAP, ADCY5, ACTR10
Prostate	20	RINL, RAPSN, RAB11FIP2, PLCD4, PIK3C2G, NGEF, LY96, LAT, KCNMB3, INPP5E, IL9R, IL27, IGLL5, GNG3, FGF22, DAB1, COG6, CNTF, ARR3, AKAP5
Kidney (KICH)	12	**SIKE1**, ZWINT, VAMP4, USP4, SNAP29, MTMR4, LMO4, EPB41L1, DYNC1LI1, COG2, ATG12, ATG10
Bladder	3	SOCS4, PPP4R4, MYH2
Liver	3	**SORBS1**, GOLGA5, CACNA1D

Hits marked in bold show high significance change in their mRNA expression (|log_2_FC|>2 and *P *< .001), while those underlined are Rab GTPases.

## 4 Discussion

PPINs are a powerful tool in understanding the mechanisms of complex biological processes. In this study, we demonstrate the utility of PPINs by providing an analysis sequence that can be used to study any set of proteins and their contribution towards a biological event of interest. Based on the Shannon diversity index (Zitnik *et al.* 2019), we introduce *resnet*, which is a useful tool for assessing network resilience of a biological or any other type of network. The Python module *resnet* assesses network resilience by removing random nodes. This opens the way to incorporate other methods of dismantling networks (Mirzasoleiman and Jalili 2011) that induce non-random, targeted systemic failure. These include removing nodes based on their centrality within a network, their degree of connectivity and edge properties.

When identifying biomarkers or therapeutic targets, understanding the resilience of the biological system is important. Upon critical failure, a resilient network will gradually degrade in function instead of suddenly collapsing as a weak network would, owing to its high redundancy and flexibility in rerouting functions (Barabási and Oltvai 2004, Kitano 2007). As such, identifying potential biomarker nodes within a resilient network will likely require a different approach compared to that used for weaker networks. Within our analysis sequence, we used the metric resilience to choose an optimal size of the Rab network for differential expression analyses. Additionally, network density was taken into consideration; dense networks are essential for maintaining the robustness and adaptability of biological systems. However, they may introduce ‘noisy’ interactions and crosstalk, where signals from one pathway inadvertently interfere with another (Stelling *et al.* 2004, Raser and O’Shea 2005). Crosstalk across signalling pathways, as well as tissue types, can lead to incorrect assessment of hits and their complex role in metastasis. We aimed to minimize the inevitable interference of crosstalk by selecting the optimal Rab network with high resilience and moderate density.

Determining the overall topology and hubs of the optimal *Rabome* highlighted several proteins with more important biological roles in the network due to their high connectivity. Of the 33 proteins identified as hubs, nine proteins were also the hubs in a network study investigating EMT (Zhao *et al.* 2015). In that study, Zhao *et al.* (2015) had constructed an EMT-related PPIN from manually curated, experimentally verified genes. The nine common hub proteins from both studies are SRC, EGFR, AKT1, MAPK3, CTNNB1, MAPK1, TP53, PTK2, and JAK2.This overlap of hubs makes the *Rabome* an interesting PPIN for the study of EMT. It is interesting to note that these common hubs, like other proteins in the optimal *Rabome*, were not added to the base network in a pure random walk (Zhang and Zhang 2023). A new protein was added based on its total connectivity to the existing Rab network (Doncheva *et al.* 2019). However, it is worth examining the Rab network expanded by other network propagation methods.

Despite playing a major role in cancer recurrence and metastasis, the exact mechanism of EMT remains only partially understood. Moreover, the heterogeneity in cancer makes it challenging to predict the outcome of treatments and identify precise prognostic markers of metastasis. For example, chemo-resistant colonies of tumours can lead to a recurrence of cancer which has a significant contribution to patient mortality (Lim *et al.* 2022). Of the 10 cancer types included in this study, primary tumours at lung, stomach, and pancreas are most likely to metastasize to other sites at diagnosis (Abdelazeem B *et al.* 2022). Oncogenes and their interactors generally tend to be researched more due to their pathogenic implications. Hence, most hits had some previous links to cancer incidence or in a few cases, metastasis.

No evidence was found to explain the upregulation of RAB3B and RAB6B in renal metastasis. However, in addition to being a potential target for CAR-T Cell therapies (Li *et al.* 2022), the expression levels of RAB3B influence the progression of gliomas (Luo *et al.* 2021), metastasis and chemoresistance in cancer stem-like sphere cells from a human hepatoma cell line (Tsunedomi *et al.* 2022). While RAB6B has been linked to poor prognosis in breast cancer patients (van’t Veer *et al.* 2002), it is also a promising prognostic marker of hepatocellular carcinoma (Peng *et al.* 2022), and further *in vitro* studies are needed to explore its prognostic potential in renal metastasis as per our results. Despite its role in cell migration (Neumann and Prekeris 2023), we could not find direct evidence of RAB40A in stomach cancer metastasis. However, it is interesting to note that RAB40AL, a closely related isoform of RAB40A, is linked to Martin-Probst Syndrome and lung cancer cell migration (Neumann and Prekeris 2023). Other than the hits (RAB3B, RAB6B and RAB40A), three Rabs were found amongst the EMT-proteins (RAB22A, RAB43 and RAB41). A large number of Rabs listed as candidates were eliminated during the MaxLink filters (RAB1AB/11AB/8AB/4AB/6A/39AB/40BC/3AB/27AB/2A/5A/7A/33B/9A/28/10/14/21/13/25/12/15/31/34/30/23/24/18). The other Rabs not neighbouring the EMT-proteins were not considered for the MaxLink analysis. However, it is worth noting that RAB6A is involved in NOTCH signalling and RAB23 in Hedgehog signalling, making them the only Rabs in EMT-associated pathways from the pool of eliminated candidate Rabs. Moreover, Rabs themselves appeared less often at the outputs of various MaxLink filters, compared to their interacting proteins. Although it may seem as if Rabs had become less pertinent during the analyses, it is their wide-ranged function in membrane trafficking that allows for a diverse interaction network, the Rab network, to be investigated for EMT across multiple cancer types.

The optimal *Rabome* was examined for predictive markers of EMT by assessing the mRNA levels. However, a possible limitation of our analysis approach could arise in cases of weak linkage between mRNA abundance and the level of protein expression. Since mRNA and protein expression are not directly correlated, further studies are needed to factor in the role of transcription factors, and other regulatory proteins associated with protein translation and modification. Nevertheless, the analysis approach that we present lays a foundation for assessing the contribution of given proteins towards a biological event of interest. We believe that the Rab PPIN can be studied further for its role in cancer recurrence and hallmarks, and our reported hits can be further investigated in *in vitro* models for their role as stable predictive markers for cancer metastasis. In conclusion, by combining large-scale network analyses with differential expression studies, we highlight the utility of PPINs in determining potential predictive markers of cancer metastasis.

## Supplementary Material

vbae200_Supplementary_Data
