# Supplementary material for: Exploring the role of the Rab network in epithelial-to-mesenchymal transition
Source: Bioinform Adv. 2024 Dec 14;5(1):vbae200. doi: 10.1093/bioadv/vbae200 (PMC11684074; doi:10.1093/bioadv/vbae200)
Supplement: vbae200_Supplementary_Data [file vbae200_supplementary_data.zip › F1.docx]

**Exploring the role of the Rab network in Epithelial to Mesenchymal Transition**

Unmani Jaygude^1,2^, Graham M Hughes^1^ *†, Jeremy C Simpson^1,2^ *†

^1^School of Biology and Environmental Science, University College Dublin, Ireland

^2^Cell Screening Laboratory, School of Biology and Environmental Science, University College Dublin, Ireland

*Authors contributed equally

†Corresponding authors: Graham M Hughes, Jeremy C Simpson

ORCID: U.J, 0009-0002-3615-6288; G.M.H, 0000-0003-3088-345X; J.C.S, 0000-0002-7956-7805;

**Sections**

| **Section No.** | **Section Tag** | **Title** |
| --- | --- | --- |
| 1 | S1 | Expanding *Rabome* and changing topology |
| 2 | S2 | Hub Proteins of Optimal *Rabome* |
| 3 | S3 | Testing *resnet* |
| 4 | S4 | Enrichment Analysis of optimal *Rabome* |
| 5 | S5 | MaxLink- Degree filter |
| 6 | S6 | mRNA expression datasets |

**S1. Expanding *Rabome* and changing topology**

**Table 1.** Network properties after expanding the base network from STRING database. All the properties normalised in the range of [0, 1] are depicted in blue.

| **Added nodes** | **Network Size** | **Mean number of neighbours** | **NORM-Mean number of neighbours** | **Characteristic Path length** | **NORM-Characteristic Path length** | **Clustering coefficient** | **NORM-Clustering coefficient** | **Network Density** | **NORM-Network Density** | **Network heterogeneity** | **NORM-Network heterogeneity** | **Network Centralisation** | **NORM-Network Centralisation** | **Ɣ (Scale-free property)** | **NORM-Ɣ (Scale-free property)** | **Resilience (100 Bins)** | **NORM-Resilience (100 Bins)** | **NORM-Resilience (200 Bins)** | **Resilience (200 Bins)** |
| --- | --- | --- | --- | --- | --- | --- | --- | --- | --- | --- | --- | --- | --- | --- | --- | --- | --- | --- | --- |
| 0 | 421  (Base) | 8.679 | 0 | 3.09 | 1 | 0.31 | 0 | 0.021 | 0.391304 | 1.115 | 1 | 0.163 | 0.347518 | 1.234 | 1 | 0.411867058 | 0 | 0 | 0.411867058 |
| 25 | 446 | 11.726 | 0.062092 | 2.937 | 0.643357 | 0.352 | 0.281879 | 0.026 | 0.608696 | 1.102 | 0.954386 | 0.161 | 0.333333 | 1.115 | 0.62766 | 0.419688132 | 0.139737771 | 0.139480474 | 0.41966895 |
| 50 | 471 | 13.843 | 0.105233 | 2.879 | 0.508159 | 0.386 | 0.510067 | 0.029 | 0.73913 | 1.07 | 0.842105 | 0.154 | 0.283688 | 1.067 | 0.477472 | 0.423600577 | 0.209640756 | 0.210007637 | 0.423613913 |
| 75 | 496 | 15.266 | 0.134231 | 2.837 | 0.410256 | 0.372 | 0.416107 | 0.031 | 0.826087 | 1.019 | 0.663158 | 0.16 | 0.326241 | 1 | 0.267835 | 0.428539719 | 0.297887542 | 0.29710619 | 0.428485803 |
| 100 | 521 | 16.392 | 0.157177 | 2.82 | 0.370629 | 0.379 | 0.463087 | 0.032 | 0.869565 | 0.83 | 0 | 0.154 | 0.283688 | 1.04 | 0.392991 | 0.431259256 | 0.346477052 | 0.347957656 | 0.431330199 |
| 125 | 546 | 17.667 | 0.183159 | 2.803 | 0.331002 | 0.389 | 0.530201 | 0.032 | 0.869565 | 0.962 | 0.463158 | 0.152 | 0.269504 | 1.014 | 0.31164 | 0.433396789 | 0.384667986 | 0.385762187 | 0.433444809 |
| 150 | 571 | 18.851 | 0.207287 | 2.789 | 0.298368 | 0.398 | 0.590604 | 0.033 | 0.913043 | 0.941 | 0.389474 | 0.143 | 0.205674 | 0.98 | 0.205257 | 0.435571903 | 0.423530365 | 0.424248323 | 0.435597545 |
| 175 | 596 | 19.775 | 0.226117 | 2.782 | 0.282051 | 0.405 | 0.637584 | 0.033 | 0.913043 | 0.928 | 0.34386 | 0.139 | 0.177305 | 1 | 0.267835 | 0.437347373 | 0.455252378 | 0.454969776 | 0.437315961 |
| 200 | 621 | 20.576 | 0.24244 | 2.783 | 0.284382 | 0.416 | 0.711409 | 0.033 | 0.913043 | 0.914 | 0.294737 | 0.135 | 0.148936 | 0.9646 | 0.157071 | 0.438637945 | 0.478310802 | 0.480220468 | 0.438728368 |
| 300 | 721 | 23.967 | 0.311542 | 2.766 | 0.244755 | 0.428 | 0.791946 | 0.033 | 0.913043 | 0.874 | 0.154386 | 0.121 | 0.049645 | 0.992414 | 0.244099 | 0.443868804 | 0.571769647 | 0.569854369 | 0.443742073 |
| 350 | 771 | 25.761 | 0.348101 | 2.759 | 0.228438 | 0.429 | 0.798658 | 0.033 | 0.913043 | 0.86 | 0.105263 | 0.132 | 0.12766 | 0.996535 | 0.256993 | 0.44570118 | 0.6045084 | 0.603511001 | 0.44562467 |
| 450 | 871 | 30.312 | 0.440842 | 2.744 | 0.193473 | 0.439 | 0.865772 | 0.035 | 1 | 0.868 | 0.133333 | 0.178 | 0.453901 | 1.001 | 0.271026 | 0.448585109 | 0.656035058 | 0.658322961 | 0.448690597 |
| 550 | 971 | 33.889 | 0.513735 | 2.727 | 0.153846 | 0.437 | 0.852349 | 0.035 | 1 | 0.896 | 0.231579 | 0.22 | 0.751773 | 0.93935 | 0.078066 | 0.451015581 | 0.699459881 | 0.69880707 | 0.450955091 |
| 650 | 1071 | 37.365 | 0.58457 | 2.693 | 0.074592 | 0.427 | 0.785235 | 0.035 | 1 | 0.906 | 0.266667 | 0.244 | 0.921986 | 0.9144 | 0 | 0.452932396 | 0.733707284 | 0.733677837 | 0.4529056 |
| 750 | 1171 | 39.87 | 0.635617 | 2.671 | 0.02331 | 0.426 | 0.778523 | 0.034 | 0.956522 | 0.908 | 0.273684 | 0.255 | 1 | 0.943903 | 0.092312 | 0.454585961 | 0.763251249 | 0.762126959 | 0.454496912 |
| 850 | 1271 | 41.638 | 0.671646 | 2.661 | 0 | 0.422 | 0.751678 | 0.033 | 0.913043 | 0.901 | 0.249123 | 0.247 | 0.943262 | 0.966145 | 0.161906 | 0.455874871 | 0.786279979 | 0.785750702 | 0.455818315 |
| 950 | 1371 | 43.152 | 0.702498 | 2.661 | 0 | 0.42 | 0.738255 | 0.031 | 0.826087 | 0.891 | 0.214035 | 0.237 | 0.87234 | 0.944863 | 0.095316 | 0.456934739 | 0.80521646 | 0.805627388 | 0.456930125 |
| 1050 | 1471 | 44.468 | 0.729316 | 2.663 | 0.004662 | 0.418 | 0.724832 | 0.03 | 0.782609 | 0.881 | 0.178947 | 0.227 | 0.801418 | 0.966813 | 0.163996 | 0.457928224 | 0.822966869 | 0.822722531 | 0.457886348 |
| 1550 | 1971 | 46.154 | 0.763674 | 2.686 | 0.058275 | 0.413 | 0.691275 | 0.023 | 0.478261 | 0.917 | 0.305263 | 0.2 | 0.609929 | 1.059315 | 0.453426 | 0.461311466 | 0.883414672 | 0.883893092 | 0.461307946 |
| 2050 | 2471 | 45.74 | 0.755237 | 2.727 | 0.153846 | 0.422 | 0.751678 | 0.019 | 0.304348 | 0.967 | 0.480702 | 0.177 | 0.446809 | 1.123361 | 0.65382 | 0.463198251 | 0.917125537 | 0.917453752 | 0.463185174 |
| 2550 | 2971 | 48.879 | 0.819204 | 2.765 | 0.242424 | 0.435 | 0.838926 | 0.016 | 0.173913 | 0.928 | 0.34386 | 0.15 | 0.255319 | 1.098778 | 0.576902 | 0.464678192 | 0.943567387 | 0.94371949 | 0.464654358 |
| 3050 | 3471 | 48.691 | 0.815373 | 2.789 | 0.298368 | 0.432 | 0.818792 | 0.014 | 0.086957 | 0.953 | 0.431579 | 0.132 | 0.12766 | 1.181364 | 0.835307 | 0.465563273 | 0.959380983 | 0.960143635 | 0.465573048 |
| 4050 | 4471 | 52.917 | 0.901492 | 2.85 | 0.440559 | 0.444 | 0.899329 | 0.012 | 0 | 0.948 | 0.414035 | 0.114 | 0 | 1.168082 | 0.793748 | 0.467193322 | 0.988504779 | 0.988280219 | 0.467146879 |
| 4550 | 4971 | 57.751 | 1 | 2.874 | 0.496503 | 0.459 | 1 | 0.012 | 0 | 0.948 | 0.414035 | 0.125 | 0.078014 | 1.16808 | 0.793742 | 0.467836705 | 1 | 1 | 0.467802429 |

**Table 2.** The normalised resilience of base network and all expanding Rab networks obtained by two binning methods (100 and 200 bins). The data for the networks in the steady zone (blue) illustrates the smaller % change in resilience for the larger networks. The smallest Rab network within the steady zone was chosen for the further analysis (highlighted in grey), on account of a less % change in resilience between its consecutive networks.

| **Added nodes to the base network** | **Network Size** | **Normalised Resilience (100 Bins)** | **Normalised Resilience (200 Bins)** | **% Difference in normalised resilience for consecutive networks (100 Bins)** | **% Difference in normalised resilience for consecutive networks (200 Bins)** |
| --- | --- | --- | --- | --- | --- |
| 0 | 421 | 0.0000 | 0.0000 | 100.00 | 100.00 |
| 25 | 446 | 0.1397 | 0.1395 | 33.34 | 33.58 |
| 50 | 471 | 0.2096 | 0.2100 | 29.62 | 29.32 |
| 75 | 496 | 0.2979 | 0.2971 | 14.02 | 14.61 |
| 100 | 521 | 0.3465 | 0.3480 | 9.93 | 9.80 |
| 125 | 546 | 0.3847 | 0.3858 | 9.18 | 9.07 |
| 150 | 571 | 0.4235 | 0.4242 | 6.97 | 6.75 |
| 175 | 596 | 0.4553 | 0.4550 | 4.82 | 5.26 |
| 200 | 621 | 0.4783 | 0.4802 | 16.35 | 15.73 |
| 300 | 721 | 0.5718 | 0.5699 | 5.42 | 5.58 |
| 350 | 771 | 0.6045 | 0.6035 | 7.85 | 8.33 |
| 450 | 871 | 0.6560 | 0.6583 | 6.21 | 5.79 |
| 550 | 971 | 0.6995 | 0.6988 | 4.67 | 4.75 |
| 650 | 1071 | 0.7337 | 0.7337 | 3.87 | 3.73 |
| 750 | 1171 | 0.7633 | 0.7621 | 2.93 | 3.01 |
| 850 | 1271 | 0.7863 | 0.7858 | 2.35 | 2.47 |
| 950 | 1371 | 0.8052 | 0.8056 | 2.16 | 2.08 |
| **1050** | **1471** | **0.8230** | **0.8227** | **6.84** | **6.92** |
| **1550** | **1971** | **0.8834** | **0.8839** | **3.68** | **3.66** |
| **2050** | **2471** | **0.9171** | **0.9175** | **2.80** | **2.78** |
| **2550** | **2971** | **0.9436** | **0.9437** | **1.65** | **1.71** |
| **3050** | **3471** | **0.9594** | **0.9601** | **2.95** | **2.85** |
| **4050** | **4471** | **0.9885** | **0.9883** | **1.15** | **1.17** |
| **4550** | **4971** | **1.0000** | **1.0000** | **-** | **-** |

**Fig. 1** All network properties observed after expanding the network from STRING database.


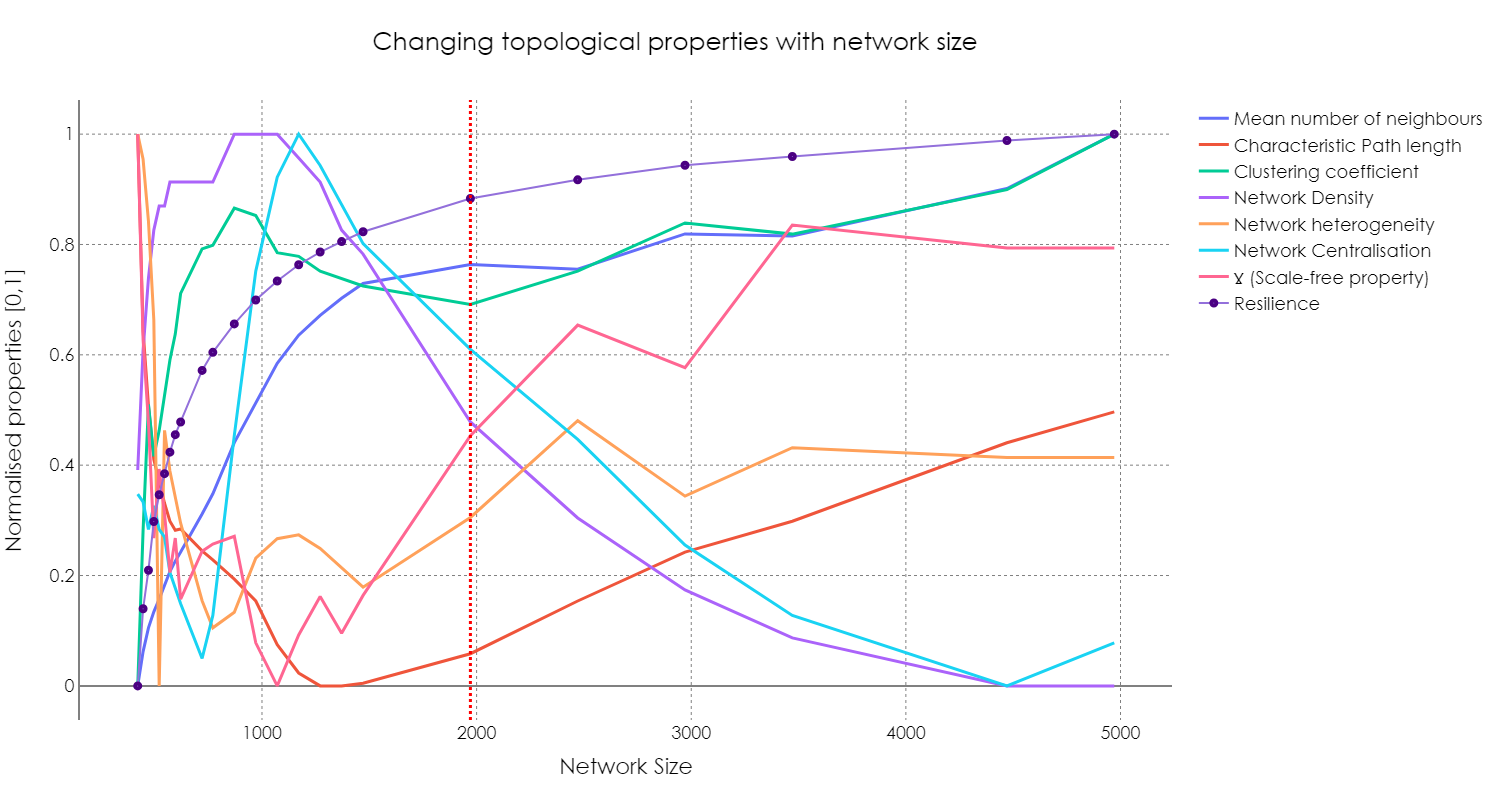


**S2. Hub Proteins of Optimal *Rabome***

**Table 3.** 33 Nodes (degree >186) identified as hubs from the optimal *Rabome*. The cut-off for defining hubs was set from the elbow of the degree distribution curve. The elbow of the curve was calculated using the method *interp1d* from the *Python* package *kneedle*. Highlighted hubs (blue) are also the hubs in a network study directed towards EMT (Zhao et al. 2015).

| **Degree** | **Protein** |
| --- | --- |
| 440 | SRC |
| 352 | EGFR |
| 339 | AKT1 |
| 319 | HRAS |
| 312 | PIK3R1 |
| 303 | PIK3CA |
| 294 | GRB2 |
| 290 | MAPK3 |
| 273 | CTNNB1 |
| 272 | MAPK1 |
| 261 | STAT3 |
| 259 | RAC1 |
| 254 | KRAS |
| 239 | CDC42 |
| 237 | TNF |
| 236 | RHOA |
| 235 | FYN |
| 233 | TP53 |
| 232 | PTK2 |
| 231 | PTPN11 |
| 220 | NRAS |
| 219 | SHC1 |
| 216 | HSP90AA1 |
| 214 | ACTB |
| 211 | PLCG1 |
| 202 | LCK |
| 200 | IL6 |
| 197 | ITGB1 |
| 197 | RPS27A |
| 196 | EGF |
| 195 | JAK2 |
| 194 | CD4 |
| 188 | JUN |

**S3. Testing *ResNet***

Without binning, we averaged each failure rate f in the range *f* ϵ [0, 1] from 500 iterations of random node removal corresponding to that failure rate. For instance, for a network of size 471 (N), random removal of 50 nodes implies a failure rate of 0.106, i.e. 50/(N, total size of the network). For this failure rate, we randomly remove 50 nodes for 500 iterations to avoid the bias introduced by chance of eliminating a hub at first attempt. Calculating the entropy for each failure starting from 1 to N became computationally inefficient for larger N values. Hence, we attempted binning the failure rates in 100 and 200 bins instead of decimating the range *f* ϵ [0, 1] of failure rates in N parts. The % error for both the inning methods was calculated by the following.

% Error = ( (Resilience)_BINNING_ - (Resilience)_NON- BIINNED_)/ (Resilience)_NON- BIINNED_ )*100

Since the error rate for the increasingly larger networks was >0.1 we used the binned the values of failure rates over the range [0,1] for further calculation of resilience.

**Table 4.** Resilience values before and after binning by 100 and 200 units, along with the % error.

| **Added nodes during expansion** | **Network Size** | **Resilience (100 Bins)** | **Resilience (200 Bins)** | **Non-Binned values** | **% Error for 100 bins** | **% Error for 200 bins** |
| --- | --- | --- | --- | --- | --- | --- |
| 0 | 421 | 0.411867058 | 0.411867058 | 0.411869 | 0.000471509 | 0.000471509 |
| 25 | 446 | 0.419688132 | 0.41966895 | 0.419685 | 0.000746274 | 0.003824297 |
| 50 | 471 | 0.423600577 | 0.423613913 | 0.423591 | 0.002260907 | 0.005409227 |
| 75 | 496 | 0.428539719 | 0.428485803 | 0.428538 | 0.000401131 | 0.01218025 |
| 100 | 521 | 0.431259256 | 0.431330199 | 0.431214 | 0.010495021 | 0.026946945 |
| 125 | 546 | 0.433396789 | 0.433444809 | 0.433397 | 4.86852E-05 | 0.011031225 |
| 150 | 571 | 0.435571903 | 0.435597545 | 0.435572 | 2.22696E-05 | 0.005864702 |
| 175 | 596 | 0.437347373 | 0.437315961 | 0.437347 | 8.5287E-05 | 0.007097111 |
| 200 | 621 | 0.438637945 | 0.438728368 | 0.438638 | 0 | 0 |

**S4. Enrichment Analysis of optimal *Rabome***

Following were the parameters used in enrichment analysis using FLAME. The input file was a list of all the proteins in optimal *Rabome*. Four pipelines used, namely, aGOtool, gProfiler, WebGestalt, enrichR. The pathways found to be enriched from all four pipelines (Rank=4) were looked at. The combined P-value obtained from four pipelines were used as criterion for significance of enrichment of pathways with Rank 4.


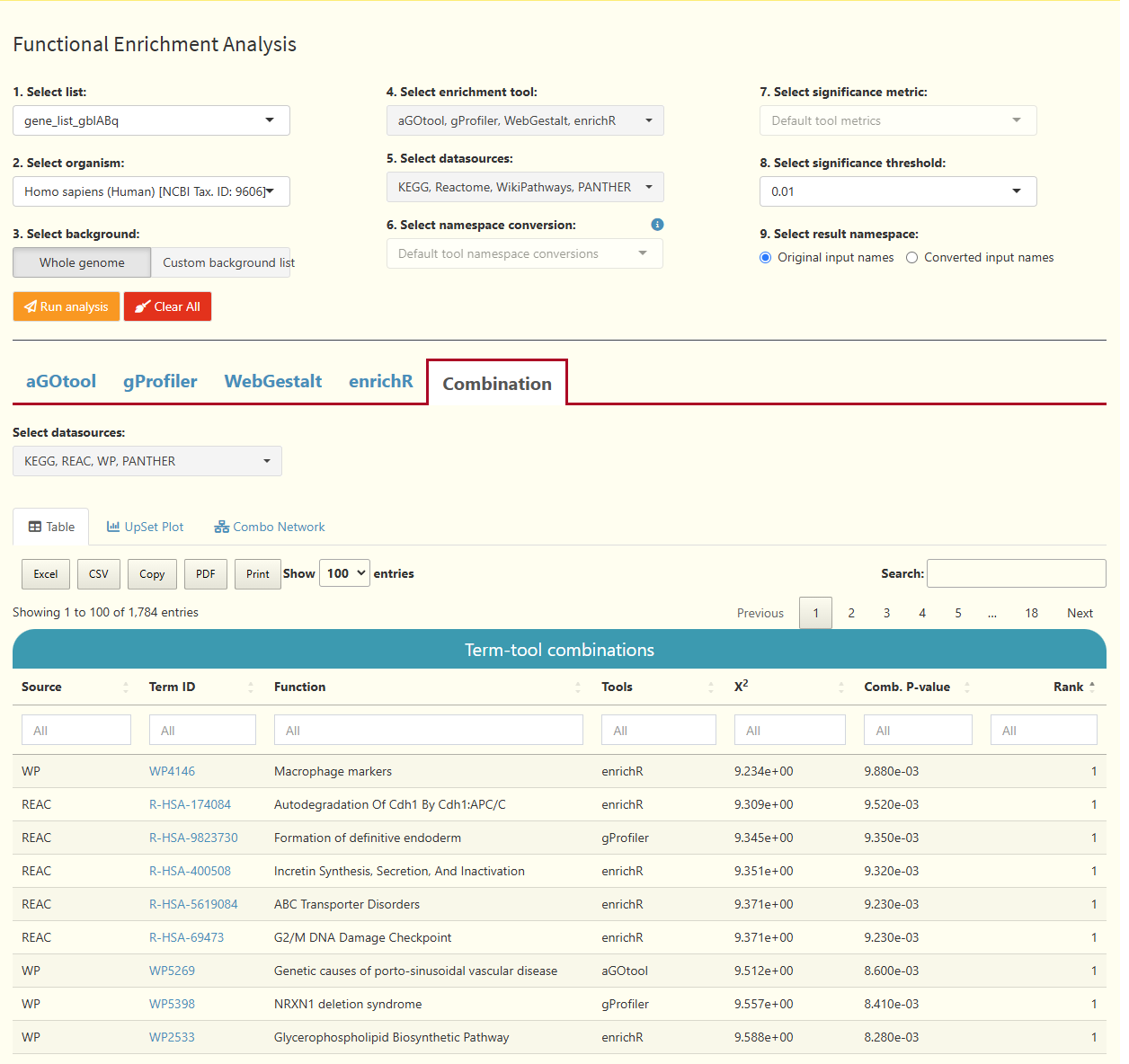


The following pathways hold rank 4 corresponding to the methods used for pathway enrichment analysis, namely, aGOtool, gProfiler, WebGestalt, enrichR. The pathways were significantly enriched (combined P-value<0.001) through the 4 aforementioned methods.

**Table 5.** Top 5 enriched pathways in *Rabome*.

| **Source** | **Term ID** | **Function** | **X2** | **Comb. P-value** |
| --- | --- | --- | --- | --- |
| REAC | R-HSA-5663205 | Infectious disease | 9.94E+02 | 3.26E-209 |
| WP | WP2059 | Alzheimer's disease and miRNA effects | 4.33E+02 | 1.72E-88 |
| KEGG | map05130 | Pathogenic Escherichia coli infection | 4.04E+02 | 2.62E-82 |
| KEGG | map04020 | Calcium signaling pathway | 3.44E+02 | 1.51E-69 |
| REAC | R-HSA-418594 | G alpha (i) signalling events | 3.26E+02 | 1.21E-65 |

**Table 6.** Pathways enriched in *Rabome* that are associated with EMT.

| **Source** | **Term ID** | **Function** | **Term enriched in other databases** | **X2** | **Comb. P-value** |
| --- | --- | --- | --- | --- | --- |
| REAC | R-HSA-9012852 | Signaling by NOTCH3 | ~ | 6.30E+01 | 1.19E-10 |
| WP | WP61 | Notch signaling pathway | Reactome, KEGG | 2.35E+02 | 2.10E-46 |
| REAC | R-HSA-5358351 | Signaling by Hedgehog | WP, KEGG | 2.04E+02 | 1.12E-39 |
| REAC | R-HSA-4791275 | Signaling by WNT in cancer | WP | 2.06E+02 | 4.33E-40 |
| WP | WP3859 | TGF-beta signaling in thyroid cells for epithelial-mesenchymal transition | ~ | 1.44E+02 | 4.05E-27 |
| REAC | R-HSA-389357 | CD28 dependent PI3K/Akt signaling | ~ | 2.35E+02 | 2.91E-46 |
| REAC | R-HSA-1643713 | Signaling by EGFR in Cancer | ~ | 2.41E+02 | 1.38E-47 |
| REAC | R-HSA-1226099 | Signaling by FGFR in disease |  | 2.59E+02 | 2.34E-51 |

**S5. MaxLink- Degree filter**

**Fig. 2** Loess fit for the connectivity of candidates to EMT-proteins vs their overall connectivity in the optimal *Rabome.* The range highlighted in red indicates the 95% confidence interval of the Loess fitting curve. The candidates within this range were filtered out.

***
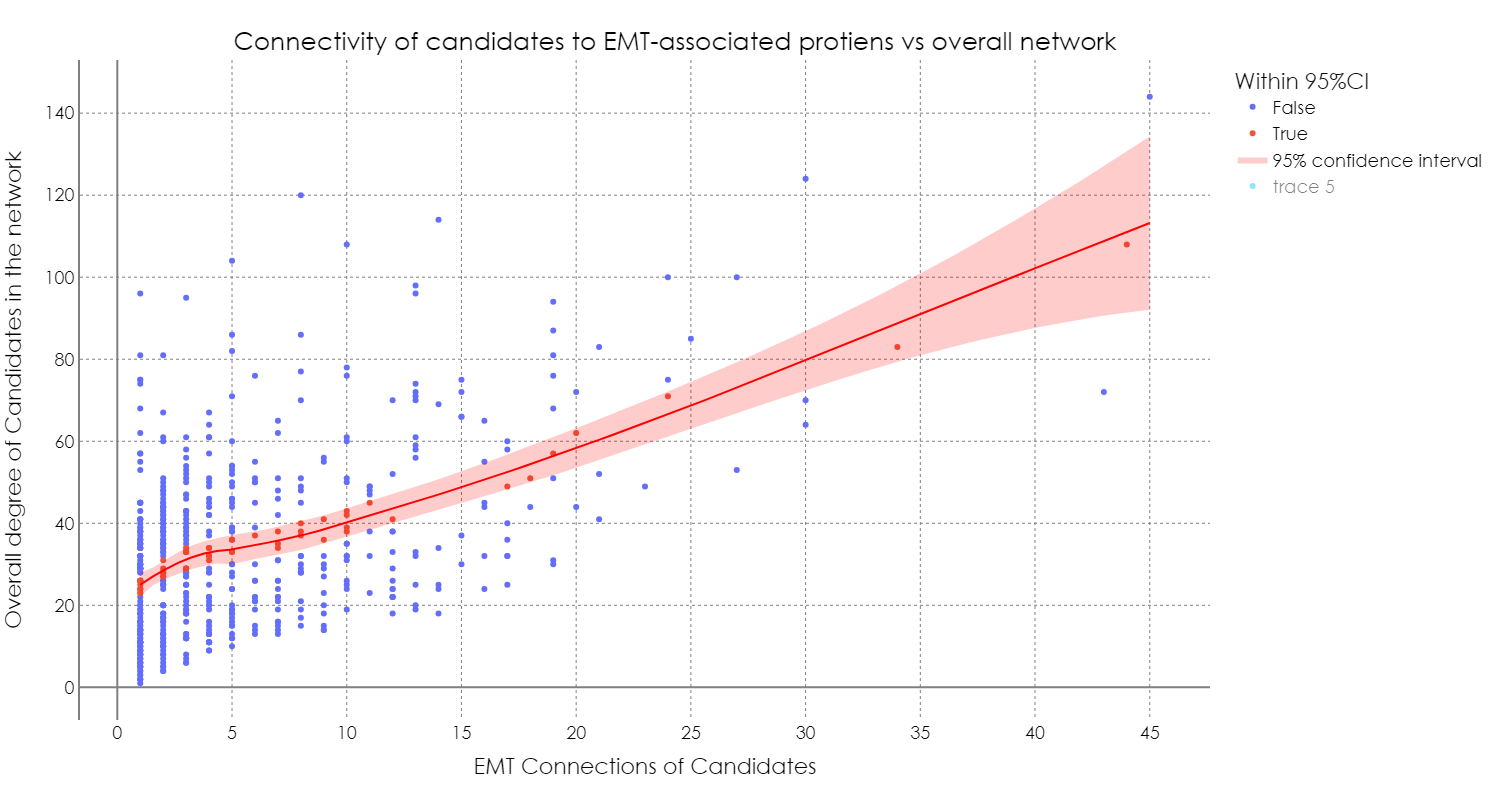
***

**S6. mRNA expression datasets**

**Fig. 3** Number of samples and their source datasets used in the differential expression analysis, respective to each cancer type and cohort (normal, primary, metastatic)

**
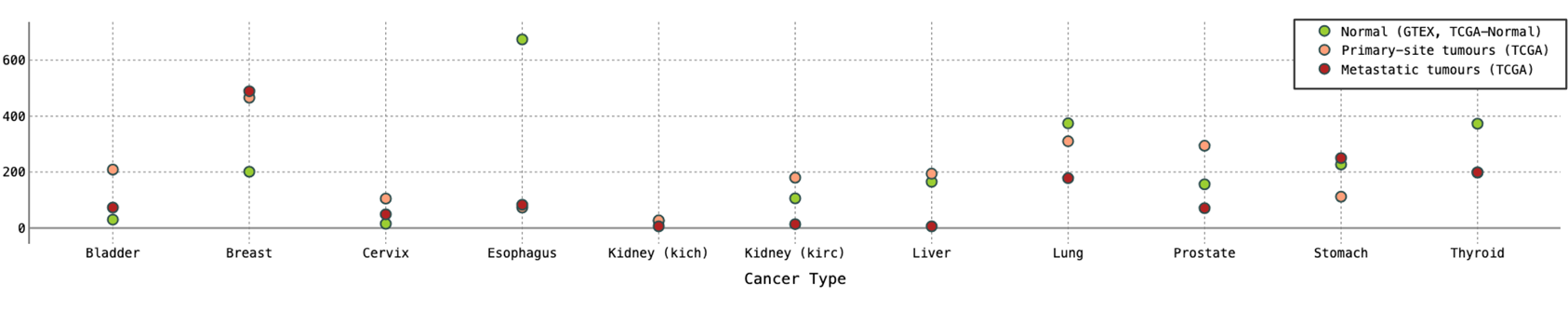
**

**References**

Zhao M, Kong L, Liu Y *et al.* dbEMT: an epithelial-mesenchymal transition associated gene resource. *Sci Rep* 2015;**5**:11459.
